# Supplementary material for: The Primary Complete Mitochondrial Genome of the Lappet Moth Brahmophthalma hearseyi (Lepidoptera: Brahmaeidae) and Related Phylogenetic Analysis
Source: Insects. 2021 Oct 28;12(11):973. doi: 10.3390/insects12110973 (PMC8620751; doi:10.3390/insects12110973)
Supplement: Supplementary file 1 [file insects-12-00973-s001.zip › insects-1376313-supplementary.pdf]

Supplementary Table S1. Universal primer sequences and condition of Long-distance PCR used to amply the mitochondrial genome of *Brahmophthalma hearseyi*

Primer name	Primer sequence	Fragment size(kb)
COI-F	GGTCAACAAATCATAAAGAT ATTG	0.70
COI-R	TAAACTTCAGGGTGACCAAAAAAT	
COⅢ-F	GTTGATTATAGACCWTGRCC	0.72
COⅢ-R	TCAACAAAATGTCARTAYCA	
ND4-F	CCAGAAGAACATAANCCRTG	0.57
ND4-R	TGAGGTTATCAACCNGARCG	
CytB-F	TATGTACTACCATGAGGACAAATATC	0.45
CytB-R	ATTACACCTCCTAATTTATTAGGAAT	
12SrRNA-F	TACTATGTTACGACTTAT	0.43
12SrRNA-R	AAACTAGGATTAGATACCC	
COI-COⅢ F	CGGATGAACAGTATACCCTCCATT	3.50
COI-COⅢ R	ATTCCGTGGAATCCTGTTGCT	
COⅢ-ND4 F	TATCAATGATGACGAGATGTATCTC	3.87
COⅢ-ND4 R	CATGTAGAGGCTCCAGTTTCA	
ND4-CytB F	CTGAAACTGGAGCCTCTACAT	2.15
ND4-CytB R	AAATGGTAATAAGAAATGGAAAGT	
CytB-12S F	AACCCCTCTTCATATTCAACCC	3.18
CytB-12S R	ACATATTGCCCGTCGCTTT	
12S-COI F	TGAACTAAAATACCGCCAAA	3.00
12S-COI R	TAAAATGGGGTCTCCTCCT	

Supplementary Table S2. Organization of the *Brahmophthalma hearseyi* mitochondrial genome.

Gene	Start	Stop	IGNc	Direction	Size	Anticodon	Start codon	Stop codon
COX1	1	1533	2	H	1533		CGA	TAA
trnL1	1536	1602	0	H	67	TAA		
COX2	1603	2284	0	H	682		GTG	T
trnK	2285	2355	0	H	71	CTT		
trnD	2356	2421	0	H	66	GTC		
ATP8	2422	2589	-7	H	168		ATC	TAA
ATP6	2583	3260	3	H	678		ATG	TAA
COX3	3264	4052	2	H	789		ATG	TAA
trnG	4055	4121	0	H	67	TCC		
ND3	4122	4475	5	H	354		ATT	TAA
trnA	4481	4550	2	H	70	TGC		
trnR	4553	4619	0	H	67	TCG		
trnN	4620	4687	49	H	68	GTT		
trnS1	4737	4802	17	H	66	GCT		
trnE	4820	4886	-1	H	67	TTC		
trnF	4886	4951	-15	L	66	GAA		
ND5	4937	6668	21	L	1732		ATT	T
trnH	6690	6757	-1	L	68	GTG		

Gene	Start	Stop	IGNc	Direction	Size	Anticodon	Start codon	Stop codon
ND4	6757	8097	17	L	1341		ATG	TAA
ND4L	8115	8399	2	L	285		ATG	TAA
trnT	8402	8464	0	H	63	TGT		
trnP	8465	8529	8	L	65	TGG		
ND6	8538	9068	5	H	531		ATA	TAA
CYTB	9074	10225	3	H	1152		ATG	TAA
trnS2	10229	10294	20	H	66	TGA		
ND1	10315	11253	1	L	939		ATG	TAA
trnL2	11255	11324	-25	L	70	TAG		
rrnL	11300	12676	0	L	1377			
trnV	12677	12742	0	L	66	TAC		
rrnS	12743	13522	-1	L	780			
A+T-rich region	13523	13977	0	H	455			
trnM	13978	14045	2	H	68	CAT		
trnI	14048	14115	-3	H	68	GAT		
trnQ	14113	14181	49	L	69	TTG		
ND2	14231	15244	-2	H	1014		ATT	TAA
trnW	15243	15310	-8	H	68	TCA		
trnC	15303	15369	1	L	67	GCA		
trnY	15371	15437	4	L	67	GTA		

IGN: intergenic nucleotides; negative numbers indicate overlapping nucleotides between adjacent genes.

Supplementary Table S3. Nucleotide composition features within *Brahmophthalma hearseyi* mitochondrial genome

		T(%)	C(%)	A(%)	G(%)	Size(bp)	A+T(%)	AT-skew	GC-skew
All genes		40.67	11.72	40.13	7.47	15442	80.81	-0.007	-0.221
rRNA genes		42.74	11.27	41.12	4.87	2157	83.87	-0.019	-0.397
tRNA genes		40.61	10.14	41.22	8.04	1480	81.82	0.007	-0.116
Protein-coding genes	All codons	45.96	10.14	33.25	10.65	11163	79.21	-0.160	0.025
	1st	38.11	9.92	36.01	15.96	3721	74.12	-0.028	0.233
	2st	48.29	16.45	21.88	13.38	3721	70.17	-0.376	-0.103
	3st	51.46	4.06	41.87	2.61	3721	93.33	-0.103	-0.217
A+T-rich region		51.87	3.30	43.74	1.10	455	95.60	-0.085	-0.500

Supplementary Table S4. Codon usage of the protein-coding genes of the *Brahmophthalma hearseyi* mitogenomes.

Codon	Count	RSCU	Codon	Count	RSCU
UUU(F)	339	1.82	UAU(Y)	187	1.91
UUC(F)	34	0.18	UAC(Y)	9	0.09
UUA(L)	502	5.2	UAA(*)	0	0
UUG(L)	12	0.12	UAG(*)	0	0
CUU(L)	43	0.45	CAU(H)	59	1.79
CUC(L)	2	0.02	CAC(H)	7	0.21
CUA(L)	20	0.21	CAA(Q)	53	1.74
CUG(L)	0	0	CAG(Q)	8	0.26
AUU(I)	427	1.85	AAU(N)	242	1.88
AUC(I)	19	0.08	AAC(N)	15	0.12
AUA(I)	245	1.06	AAA(K)	91	1.84
AUG(M)	15	1	AAG(K)	8	0.16
GUU(V)	73	2.1	GAU(D)	53	1.66
GUC(V)	1	0.03	GAC(D)	11	0.34
GUA(V)	63	1.81	GAA(E)	64	1.8
GUG(V)	2	0.06	GAG(E)	7	0.2
UCU(S)	114	2.6	UGU(C)	29	2
UCC(S)	15	0.34	UGC(C)	0	0
UCA(S)	80	1.83	UGA(W)	94	3
UCG(S)	1	0.02	UGG(W)	2	1
CCU(P)	78	2.52	CGU(R)	12	0.59
CCC(P)	10	0.32	CGC(R)	0	0
CCA(P)	36	1.16	CGA(R)	38	1.85
CCG(P)	0	0	CGG(R)	3	0.15
ACU(T)	85	2.19	AGU(S)	48	1.1
ACC(T)	12	0.31	AGC(S)	5	0.11
ACA(T)	57	1.47	AGA(R)	69	3.37
ACG(T)	1	0.03	AGG(R)	1	0.05
GCU(A)	82	2.67	GGU(G)	44	0.89
GCC(A)	8	0.26	GGC(G)	3	0.06
GCA(A)	32	1.04	GGA(G)	114	2.31
GCG(A)	1	0.03	GGG(G)	36	0.73

*: stop codon. stop codons were excluded in total codon counts.

Supplementary Table S5. Amino acid composition of 13 proteins of the *Brahmophthalma hearseyi* mitochondrial genome

Amino acid	Composition(%)	Amino acid	Composition(%)	Amino acid	Composition(%)
Ala(A)	3.31	Ile(I)	11.99	Arg(R)	1.42
Cys(C)	0.78	Lys(K)	2.66	Ser(S)	8.95
Asp(D)	1.72	Leu(L)	15.56	Thr(T)	4.17
Glu(E)	1.91	Met(M)	6.99	Val(V)	3.74
Phe(F)	10.02	Asn(N)	6.91	Trp(W)	2.58
Gly(G)	5.29	Pro(P)	3.33	Tyr(Y)	5.27
His(H)	1.77	Gln(Q)	1.64		

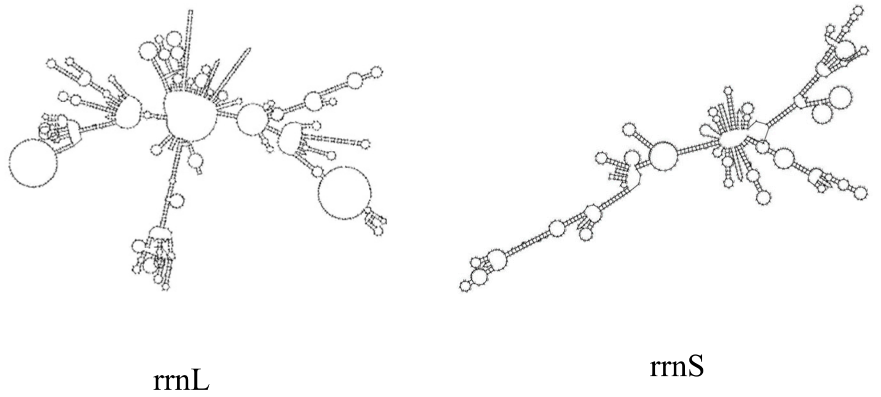

Supplementary Figure S1. Predicted secondary structure of the *Brahmophthalma hearseyi* 2 rRNA genes.

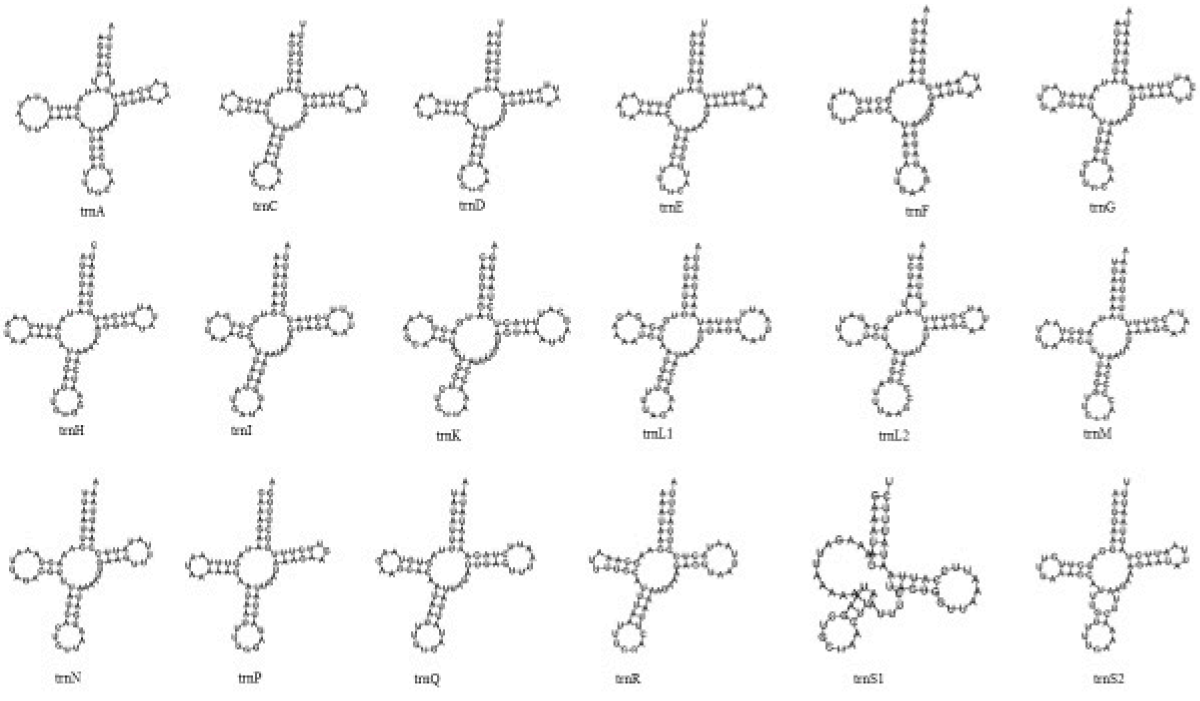

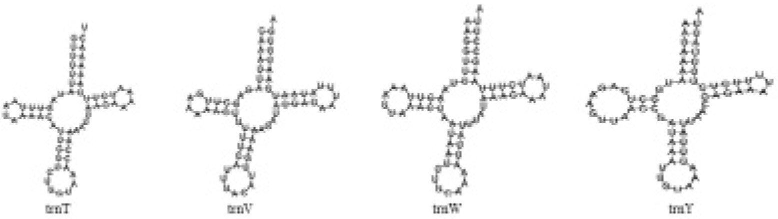

Supplementary Figure S2. Predicted secondary clover-leaf structure of the *Brahmophthalma hearseyi* 22 tRNA genes.

rrnS-
13523TATATTTATATATATATAAATATACATAGATTTTTTTTTTTTTTTTTATATTAAAATATTTATTAT
AAATTTATTTTTAATATTATATATATATATAAAATATATTTAATTATAATTTATATTAAATTATTTATTAT
AATATTATTAAATTTTTAATATTCATTTATATATATATTAAAATATTTATTATAAATTATTAAACATTGA
ATAATTTTCTTATTTTTTTTTTCATAATATTTATATTAAAACCAAAATGGCTATTTAAATTTTTATAAATT
AATAGTTTATAAAAAAATTATATAATTAATTTTAATTTATCTATAACTTATTATATTATATATATATATA
TATTATTAAATTTTTAATATAATAATAATAATAATAATAAATATTTCTTTTTTTTTTTTATTAAACCAATC
TTAATAAATTTACATATAATAATAAATAAATAAAA13977-trnM

Supplementary Figure S3. Features of the AT-rich region of *Brahmophthalma hearseyi*
